# Correction to “Influence of the Sex of Translocation Carrier on Clinical Outcomes of Couples Undergoing Preimplantation Genetic Testing”

**DOI:** 10.1002/mgg3.70071

**Published:** 2025-02-07

Zhang Z, Chen J, Zhang L, Wei R, Liu Z, Zhao D, Bi X, Liang L, Zhang X, Su D, Wu X. Influence of the Sex of Translocation Carrier on Clinical Outcomes of Couples Undergoing Preimplantation Genetic Testing. *Molecular Genetics & Genomic Medicine* 2025 Jan;13(1):e70050. https://doi.org/10.1002/mgg3.70050. PubMed PMID: 39727918.

^1^Center of Reproductive Medicine, Affiliated Children's Hospital of Shanxi & Women Health Center of Shanxi Medicine University, Taiyuan, Shanxi, China | ^2^Yikon Genomics Co., Ltd., Suzhou, China.

Correction to the affiliation.

The name “Yikon Genomics Company Ltd., Suzhou, China” of the institution was incorrect.

The name should be “Yikon Genomics Co., Ltd., Suzhou, China.”

We apologize for this error.

